# Miz1 Deficiency in the Mammary Gland Causes a Lactation Defect by Attenuated Stat5 Expression and Phosphorylation

**DOI:** 10.1371/journal.pone.0089187

**Published:** 2014-02-19

**Authors:** Adrián Sanz-Moreno, David Fuhrmann, Elmar Wolf, Björn von Eyss, Martin Eilers, Hans-Peter Elsässer

**Affiliations:** 1 Department of Cytobiology, Philipps-University Marburg, Marburg, Germany; 2 Theodor-Boveri-Institute, Biocentre, University of Würzburg, Würzburg, Germany; Sun Yat-sen University Cancer Center, China

## Abstract

*Miz1* is a zinc finger transcription factor with an N-terminal POZ domain. Complexes with Myc, Bcl-6 or Gfi-1 repress expression of genes like *Cdkn2b* (p15^Ink4^) or *Cdkn1a* (p21^Cip1^). The role of Miz1 in normal mammary gland development has not been addressed so far. Conditional knockout of the Miz1 POZ domain in luminal cells during pregnancy caused a lactation defect with a transient reduction of glandular tissue, reduced proliferation and attenuated differentiation. This was recapitulated *in vitro* using mouse mammary gland derived HC11 cells. Further analysis revealed decreased Stat5 activity in *Miz1*Δ*POZ* mammary glands and an attenuated expression of Stat5 targets. Gene expression of the Prolactin receptor (PrlR) and ErbB4, both critical for Stat5 phosphorylation (pStat5) or pStat5 nuclear translocation, was decreased in *Miz1ΔPOZ* females. Microarray, ChIP-Seq and gene set enrichment analysis revealed a down-regulation of Miz1 target genes being involved in vesicular transport processes. Our data suggest that deranged intracellular transport and localization of PrlR and ErbB4 disrupt the Stat5 signalling pathway in mutant glands and cause the observed lactation phenotype.

## Introduction

Mammary gland development occurs predominantly after birth when the mammary gland anlage starts to invade the fat pad as a tree of growing and branching ducts [1]. At this stage the end of the ducts exhibit teardrop shaped structures called terminal end buds (TEBs), which disappear when the adult virgin gland has been fully developed. The duct epithelium consists of luminal and myoepithelial basal cells. TEBs contain as an outermost layer the cap cells and as 6–10 innermost layers the so called body cells. High proliferation and balanced apoptosis in the TEB compartment drive the development of the mammary gland ductal tree from puberty to the adult virgin state. During pregnancy, hormones like estrogen, progesterone or prolactin induce a further expansion of the duct tissue as well as the formation of alveoli which almost completely displace the adipose tissue of the fat pad. In late pregnancy the synthesis of milk proteins is initiated. After weaning, the glandular tissue involutes by apoptosis and autophagy, leading to a virgin-like structure of the mammary gland.

The transcription factor Myc is important for the growth of tissues and organisms, either by controlling cell number or cell size [2], [3] and plays a pivotal role in a variety of cancers including breast cancer [4]. During pregnancy the mammary gland undergoes dramatic changes including an increase of size [5]. A conditional knockout of *Myc* in mammary gland epithelial cells during pregnancy delays the alveolar development by attenuation of cell proliferation and reduced milk production [6]. In contrast, overexpression of Myc between D12.5 to D15.5 induces a precocious lobuloalveolar development and lactation, leading to a premature mammary gland involution. This accelerated mammary gland development during pregnancy is strongly correlated with an activation of Signal Transducer and Activator of Transcription 5 (Stat5) induced by down-regulation of the Stat5 inhibitor caveolin-1 [7]. Stat5, originally described as mammary gland factor (MGF) [8], is a central regulator in mammary gland development, being mainly activated by Janus Kinase 2 (Jak2). Jak2 in turn integrates signals from different receptors like the growth hormone receptor, the estrogen receptor or the prolactin receptor, the latter being the most important one during pregnancy and lactation [5], [9]. Stat5 is expressed in the two homologous variants Stat5a and Stat5b, with Stat5a providing 70% of total Stat5 in the mammary gland [10]. Recently, it was suggested that the particular functions during mammary gland development depend on the Stat5 concentration [11]. Deletion of the largely redundant Stat5a/b variants during different stages of mouse mammary gland development prevents proper proliferation, differentiation and lobuloalveolar development during pregnancy and lactation [12]. In contrast, ectopic expression of Stat5 in mammary gland epithelial cells induces alveolar fate commitment and lactogenesis [13], [14].

The transcription factor Miz1 (Myc-interacting zinc finger protein 1; *Zbtb17*) contains 13 zinc finger motifs and a so called POZ domain (poxvirus zinc finger protein) at the N-terminus [15]. Miz1 was originally identified as a Myc binding protein, forming a repressive Myc/Miz1 complex [16], [17]. This has been shown for genes like *Cdkn2b* (encoding p15Ink4b), *Cdkn1a* (encoding p21Cip1), *Cdkn1c* (encoding p57Kip2), *Mxd4* (encoding Mad4) or *Itgb1* (encoding integrin ß1) [18]–[21]. A constitutive knockout of Miz1 is lethal at day 7.5 of embryonic development [22]. A conditional knockout of the Miz1 POZ domain in keratinocytes leads to a complex hair follicle phenotype [23] and to attenuated tumorigenesis in a skin tumor model due to p21^cip1^ deregulation [24]. In the hematopoetic system, deletion of the Miz1 POZ domain disrupts B- and T-cell development by down-regulating the IL-7 signalling pathway, most likely due to an increased expression of Socs1, an inhibitor of the Jak2/Stat5 pathway [25]–[27]. In these studies the function of Miz1 seems to be Myc-independent.

Here, we provide the first study of Miz1 function in the mammary gland epithelium. We used whey acidic protein-(Wap)-Cre recombinase to knock out the Miz1 POZ domain in luminal cells of the mammary gland during pregnancy. We observed a reduction of glandular tissue during early lactation with a corresponding lactation defect, based on reduced proliferation and attenuated differentiation. *In vitro* acinar reconstitution and differentiation assays revealed impaired proliferation, differentiation and morphogenetic capabilities in HC11 cells after Miz1 down-regulation. We provide evidence that deletion of Miz1 has pleiotropic effects on vesicular transport processes. Our results suggest that Miz1 deficiency leads to a defective prolactin receptor and ErbB4 trafficking and consequently to a perturbed activation of Stat5. In turn, this may largely account for the defects observed in *Miz1*Δ*POZ* mammary glands.

## Materials and Methods

### Mice, Genotyping and Pup Weights

*Wap-Cre* animals [28] were crossed with *Miz1^lox/lox^* mice [23] in order to conditionally knockout the POZ domain of Miz1 in luminal mammary epithelial cells during late pregnancy and lactation (Fig. S2). Prior to mating, both lines were backcrossed six generations to a 129S2/SvHsd background. In the present study, *Wap-Cre+ Miz1^+/+^* animals are referred to as control (*Ctr)* and *Wap-Cre+ Miz^lox/lox^* as *Miz1ΔPOZ*. Experiments with mice followed the German Animal Protection Law (Tierschutzgesetz) and were approved by the experimental animal local authorities (Regierungspraesidium Giessen). Project reference number: V54-19c20/15cMR20/10 Nr. A 1/2010. Genotyping of the animals was performed as described before [23], [24] using the REDExtract-N-Amp™ Tissue PCR Kit (XNATR; Sigma). Primer sequences are available in Table S1. Pup weights represented in Fig. 2 were measured every 3 days. The number of progeny was standardized to 6 pups after birth and for each genotype pups from 6 mothers were monitored. *Wap-Cre* females were mated at 65 dpp for 1^st^ pregnancy/lactation analysis and 2 weeks after completion of the 1^st^ lactation for the study of the 2^nd^ pregnancy/lactation. For pregnancy samples, mating plugs were checked every day and mammary dissection was performed at the indicated time points.

### Histology, Whole-mounts, Fat Quantification and Lipid Staining

9^th^ inguinal mammary glands [29] were dissected and fixed overnight at 4°C using freshly prepared 3.7% paraformaldehyde (PFA) in PBS. Samples were stored in 70% Ethanol at 4°C until paraffin embedding. H&E and immunostainings were performed in 3–4 µm sections using standard protocols. For whole-mount staining, 4^th^ inguinal mammary glands were spread on a glass slide, fixed in Carnoy’s solution for 4 hours at room temperature, hydrated, stained in carmine alum overnight, dehydrated and cleared in xylene [29]. The percentage of fat was calculated by estimating the area occupied by fat divided by the total mammary area on 10x H&E stained sections using ImageJ 1.43u software. 10 pictures per animal were quantified. Sudan III lipid staining was performed on lactation day 6 cryosections (1^st^ pregnancy) following standard protocols. Briefly, sections were fixed in 4% PFA for 30 minutes at 4°C, washed with 50% Ethanol and incubated with a 0.3% Sudan III-solution in 70% ethanol for 25 minutes. Then, sections were washed again with 50% Ethanol, counterstained with H&E and mounted in Mowiol.

### Immunohistochemistry and Immunofluorescence

3.7% PFA-fixed, paraffin-embedded tissue sections were mounted on polylysine slides, de-waxed and stained using standard procedures. Sections were incubated overnight at 4°C with the following primary antibodies against: Miz1 [30], Ki67 (VP-K452, Vector Laboratories; 1∶100 for *in vivo* and 1∶50 for acini stainings), PSTAT5 (71-6900; Tyr694; Invitrogen; 1∶25), Myc (ab32072; Abcam; 1∶100), Cre (kindly provided by Dr. Christoph Kellendonk; 1∶1500), prolactin receptor (M170; Santa Cruz; 1∶150), ErbB4 (C-18; Santa Cruz; 1: 200), Cleaved Caspase-3 (9664; Cell Signalling; 1∶200 for *in vivo* and 1∶100 for acini stainings) and rabbit anti-milk serum (kindly provided by Prof. Nancy E. Hynes; 1∶5000). Antigen retrieval was performed with either 10 mM Tris/1 mM EDTA (pH 9) or 10 mM citrate buffer (pH 6) for 15–30 minutes on a steamer (except for the fluorescent milk proteins and the prolactin receptor stainings where no antigen retrieval is required). 3-amino-9-ethylcarbazole (AEC) Substrate Kit (00-2007; Invitrogen) was used for all immunoperoxidase stainings. For Miz1 staining, the Mouse on Mouse Immunodetection Kit (BMK-2202; Vector Laboratories) was utilized following manufacturer’s instructions. For immunofluorescence, Alexa Fluor secondary antibodies (A11008, A21235, A11010; Molecular Probes) were used. Nuclei were visualized with 0.7 µg/ml Hoechst 33342 (14533; Sigma) and 10 µg/ml Phalloidin-TRITC (P1951; Sigma) were used for actin filaments staining in acini. Light microscopy pictures were taken with a Leitz Diaplan microscope equipped with a MicroPublisher 3.3 RTV camera (Q-Imaging) and immunofluorescence pictures were captured with a BX61 Olympus microscope assembled with a F-View digital camera (Soft Imaging System).

### Quantification of Ki67 Staining and TUNEL Assay

The percentage of Ki67 positive cells on lactation day 6 (1^st^ pregnancy) was calculated counting Ki67 positive cells among all epithelial cells in representative 25x pictures (more than 1000 cells per animal were scored). The TUNEL assay was performed according to manufacturer’s instructions (DeadEnd Fluorometric TUNEL System, Promega). TUNEL positive cells, rare in both *Ctr* and *Miz1ΔPOZ* animals, were quantified in 10 pictures per animal (20x magnification) on lactation day 1 and 6 samples (1^st^ pregnancy). ImageJ 1.43 u software was used for both quantifications.

### Ultrastructural Analysis

For transmission electron microscopy tissue was fixed in a mixture of 2.5% glutaraldehyde, 2.5% paraformaldehyde and 0.05% picric acid in 67 mM cacodylate buffer (pH 7.4) according to Ito und Karnovsky [31]. Postfixation was performed in 1% osmium tetroxide followed by an overnight incubation with 0.3% uranyl acetate dissolved in 50 mM maleate buffer (pH 5.0). Samples were embedded in Epon according to standard procedures. Thin sections were contrasted with lead citrate and examined with a Zeiss EM 109S electron microscope.

### Western Blotting

Snap frozen thoracic mammary glands were homogenized using the Ultra Turrax T25 Homogenizer (Ika, Staufen, Germany) and lysed in ice cold RIPA buffer (150 mM sodium chloride, 1% Triton X-100, 0.5% sodium deoxycholate, 0.1% SDS and 50 mM Tris pH 8) containing protease (P8340; Sigma) and phosphatase (P5726; Sigma) inhibitor cocktails and benzonase nuclease (70664-3; Novagen). After overnight incubation of the samples at 4°C, lysates were centrifuged for 15 minutes/12000 rpm/4°C to remove cellular debris and supernatants were subsequently stored at −20°C. Protein concentrations were measured by the BCA protein assay (B9643; Sigma) and 20–60 µg of samples were resolved by SDS-PAGE (8–15% gels). The primary antibodies used were the following: Miz1 (10E2; 1∶400), Actin (A2103, Sigma; 1∶1000), rabbit anti-milk serum (1∶5000), β-Casein (S-15, Santa Cruz; 1∶200), α-Tubulin (62204, Thermo Scientific; 1∶5000), Stat5a/b (C-17, Santa Cruz; 1∶250), pStat5a/b (#05–495, Tyr694/699, Millipore; 1∶500) and Cytokeratin 18 (C-04, Abcam; 1∶1000). All primary antibody incubations were performed at 4°C overnight except for the rabbit anti-milk serum, which was done at room temperature for 1 hour [32]. The latter conditions were also applied for all HRP-conjugated secondary antibody (Biorad) incubations and the bound antibodies were visualized using the SuperSignal West Dura Substrate (#34075; Thermo Scientific). Bands were scanned from the films and intensities were measured with ImageJ [33]. Values corrected for loading were expressed as fold change of a control condition arbitrarily set to 1.

### RNA Isolation, Semi-quantitative and Quantitative PCR

Thoracic mammary glands were dissected, cut in small pieces (maximum 0.5 cm in all dimensions) and stored in RNAlater (R0901; Sigma) till homogenization in TRI Reagent (T9424; Sigma). RNA was extracted, DNase treated (740963; Macherey-Nagel) and cleaned-up using a NucleoSpin RNA column-based kit (740948; Macherey-Nagel) according to manufacturer’s instructions. 1 µg of total RNA was reverse transcribed with the RevertAid First Strand cDNA Synthesis Kit (#K1622; Thermo Scientific) using random hexamer primers. Semi-quantitative PCR was performed with a Personal Cycler (Biometra, Göttingen, Germany) following standard procedures. For quantitative PCR, SYBR Green-based real-time polymerase chain reactions (AB1167; Thermo Scientific) were run in triplicate using a Mx3005P qPCR System (Stratagene, Heidelberg, Germany). Gene expression was normalized to GAPDH and analysed by the comparative cycle threshold method (ΔΔCt). For each assay, gene expression in one of the *Ctr* animals was arbitrarily set to 1 and the relative fold change expression was calculated for the rest. ‘No template’ controls (NTC) were included in each run and product specificity was verified by dissociation curve analysis. All primer sequences are available in Table S1.

### Transfection and Retroviral Infection

Phoenix packaging cells (Orbigen, San Diego, CA) were transfected with 30 µg of *shscr* or *shMiz1* expression vectors [34] by the calcium phosphate method using standard protocols. HC11 cells were infected with retroviral supernatants in the presence of 4 µg/ml of polybrene and then cells were selected with 2 µg/ml of puromycin. Histone H2B-GFP infected HC11 cells were used to estimate the efficiency of infection and to determine the end of the antibiotic selection.

### HC11 Cell Differentiation and Acini Formation

HC11 mammary epithelial cells [35] were routinely cultured in complete growth medium: RPMI 1640, 10% FBS, 60 µg/ml gentamicin, 2 mM glutamine (all from PAA), 20 µg/ml insulin from bovine pancreas (I6634; Sigma) and 10 ng/ml of EGF (E4127; Sigma). To induce competence to differentiate, 2-day confluent HC11 cells were incubated for 48 hours in medium without EGF and with low serum (2%). To induce differentiation, competent cells were incubated with DIP medium based on RPMI 1640 and supplemented with 2% FBS, 60 µg/ml gentamicin, 2 mM glutamine, 10 µg/ml insulin, 1 µM dexamethasone (D4902; Sigma) and 5 µg/ml of prolactin from sheep pituitary gland (L6520; Sigma).

Three-dimensional acini forming culture of HC11 cells was performed as described elsewhere [36]–[38]. Briefly, 24-well plates (662160; Greiner Bio-One) coated with 40 µl Cultrex basement membrane extract (BME) Growth Factor Reduced (15.76 mg/ml batch; Trevigen) per well on sterilized 13 mm coverslips were used. Coated coverslips were allowed to solidify for at least 30 minutes at 37°C. HC11 cells were seeded at a concentration of 25.000 cells/ml in 3D medium: RPMI 1640 supplemented with 2% FBS, 60 µg/ml gentamicin, 2 mM glutamine, 10 µg/ml insulin, 5 ng/ml of EGF and 2 µg/ml of puromycin (P8833; Sigma) for selection containing 2% Cultrex. 3D medium was replaced every 4 days using a total volume of 1 ml per well. Images were captured using a confocal microscope TCS SP2 AOBS (Leica, Wetzlar, Germany) and analysed with LAS AF lite freeware (Leica Microsystems).

### Microarray Analysis

RNA integrity (RQI>9 in all samples) and concentration were assessed with the Experion automated electrophoresis station (Bio-Rad). A mouse genome Agilent-028005 array was used for the analysis of gene expression of 1^st^ pregnancy lactation day 6 *in vivo* samples (n = 4 per genotype). The resulting intensity values for the red and green channels were normalized using the lowest method within the limma package in R/BioConductor [39], [40]. Regulated probes were selected on the basis that the logarithmic (base 2) average intensity value (A-Value) was ≥5. Array data are available in ArrayExpress under the accession code E-MTAB-1718.

### ChIP-Seq and Gene Set Enrichment Analysis (GSEA)

10 µg of Miz1 antibody (Santa Cruz, C-19) were incubated with 100 µl Protein G Dynabeads (Invitrogen) in a volume of 1 ml PBS +5 mg/ml BSA at 4°C on a rotating wheel. The beads were collected on a magnetic device and resuspended in 100 µl PBS +5 mg/ml BSA per ml chromatin. The coupled antibody was incubated with the chromatin from 5×10^7^ MDA-MB-231 cells at 4°C on a rotating wheel o/n. The beads were successively washed with 1 ml Sonication buffer: 50 mM Hepes pH 7.9, 140 mM NaCl, 1 mM EDTA, 1% Triton X-100, 0.1% Na-deoxycholate, 0.1% SDS, 0.25 mM PMSF, protease inhibitor cocktail (Sigma); with 1 ml high salt buffer (sonication buffer with 500 mM NaCl) and with 1 ml LiCl wash buffer: 20 mM Tris, pH 8.0, 1 mM EDTA, 250 mM LiCl, 0.5% NP-40, 0.5% Nadeoxycholate, 0.25 mM PMSF and protease inhibitor cocktail (Sigma). Finally, the chromatin was eluted with 50 mM Tris, pH 8.0, 1 mM EDTA, 1% SDS, 50 mM NaHCO3 at 65°C for 30 minutes. The eluted chromatin was decrosslinked, Proteinase K digested and EtOH precipitated according to standard procedures. The precipitated DNA was resuspended in 30 µl H_2_O and used for Illumina library preparation.

After end repairing, an A-nucleotide was added to the 3′end of the input and precipitated DNA. Subsequently, Illumina adaptors were ligated and the DNA-mixture was purified from a 1% agarose gel (175–225 bp size fraction). After DNA extraction (gel extraction Kit, Qiagen), the library was enriched by 18 PCR cycles and controlled and quantified with the Experion-system (BioRad). 36 bases input and Miz1-ChIP libraries were sequenced on an Illumina GAIIx sequencer. Only reads passing the internal Illumina raw data-filter were aligned to the precompiled human reference genome with BOWTIE [41]. Peaks were called by MACS [42] using data from the input sample as control and determining a p-value of −10^10^.

We generated a gene set consisting of the 100 direct target promoters with the highest tag counts in their respective peak. This gene set was used for the GSEA analysis which was performed using default settings.

### Statistics

All comparisons between *Ctr* and *Miz1ΔPOZ* animals were analysed by two-tailed Student′s t-tests. A two-way ANOVA followed by Bonferroni’s post-hoc test for multiple pairwise comparisons was employed for pup weight analysis. All statistical tests were performed with Prism 5.0 software (GraphPad). *P-values*: NS (*p*>0.05); * (*p = *0.01–0.05); ** (*p = *0.001−0.01); *** (*p*<0.001). Data are shown as mean ± s.d.

## Results

### Miz1 Expression in Mammary Gland Epithelial Cells

**Figure 1:**
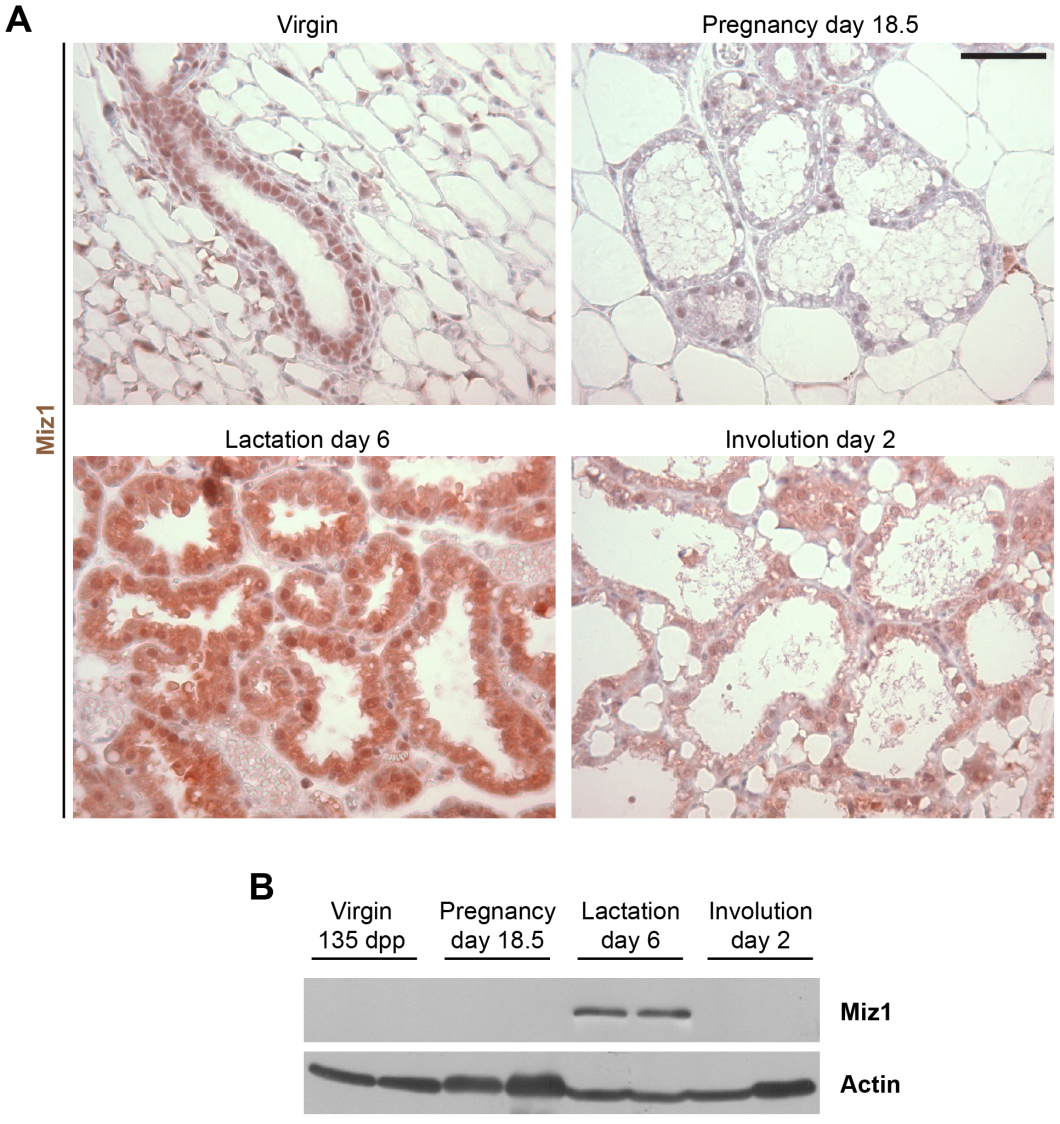
Miz1 expression in the mammary gland epithelium at different developmental stages. (**A**) Immunocytochemistry revealed a nuclear expression of Miz1 in the virgin gland as well as during pregnancy and involution. During lactation also a cytoplasmic staining was observed in addition to the nuclear stain. (**B**) Western blot analysis of Miz1 expression exhibited a strong Miz1 expression during lactation while the protein could only occasionally be detected in virgin gland. Scale bar in A: 50 µm.

Immunohistochemical stainings of Miz1 in the virgin mammary gland, during pregnancy and involution, detected nuclear Miz1 in the cells of the mammary gland ducts and alveoli, while during lactation also the cytoplasm was stained (Fig. 1A). When analysed by Western blots, Miz1 was hardly detected in virgin tissue and in glands from day 18.5 of pregnancy. In contrast, there was a strong increase of Miz1 expression related to the transition from pregnancy to lactation and Miz1 levels stayed elevated through the lactation period (Fig. 1B and unpublished data for lactation day 1 and 10). In turn, the transition from lactation to involution was correlated with a decrease of Miz1 back to levels observed during pregnancy or in the virgin gland (Fig. 1B).

In contrast, Myc expression has been shown to be high in early pregnancy until day 12.5 of gestation and decreasing to baseline levels until day 18.5 [7]. Our own immunohistochemical analysis confirms this notion (Fig. S1). Taken together, our data show that lactation is associated with a high expression of Miz1 and that the expression of Myc is regulated in an opposing trend.

### Generation of Mice Carrying a Deficient Allele of the Miz1 Gene

In order to generate a Miz1 loss-of-function mutant in luminal mammary gland cells, we used a conditional knockout mouse model, in which exons 3 and 4 of *Zbtb17*, encoding the Miz1 POZ domain, are flanked by loxP sites (Fig. S2A) [23], [25]. These mice were crossed to a transgenic mouse strain expressing the Cre recombinase under the promoter of the whey acidic protein (Wap) [28]. *Wap-Cre* is expressed in the mammary gland epithelium after day 14.5 of pregnancy [6]. In line with this notion, a PCR detecting the recombined Miz1 gene revealed a weak first signal at day 14.5 which increased during further pregnancy and lactation (Fig. S2B). Moreover, we observed Cre expression at day 18.5 of pregnancy and day 1 of lactation by an immunohistochemical approach (Fig. S2C and D). At this time point Cre recombinase was visible in almost all nuclei of the luminal cells of the glandular tissue.

**Figure 2:**
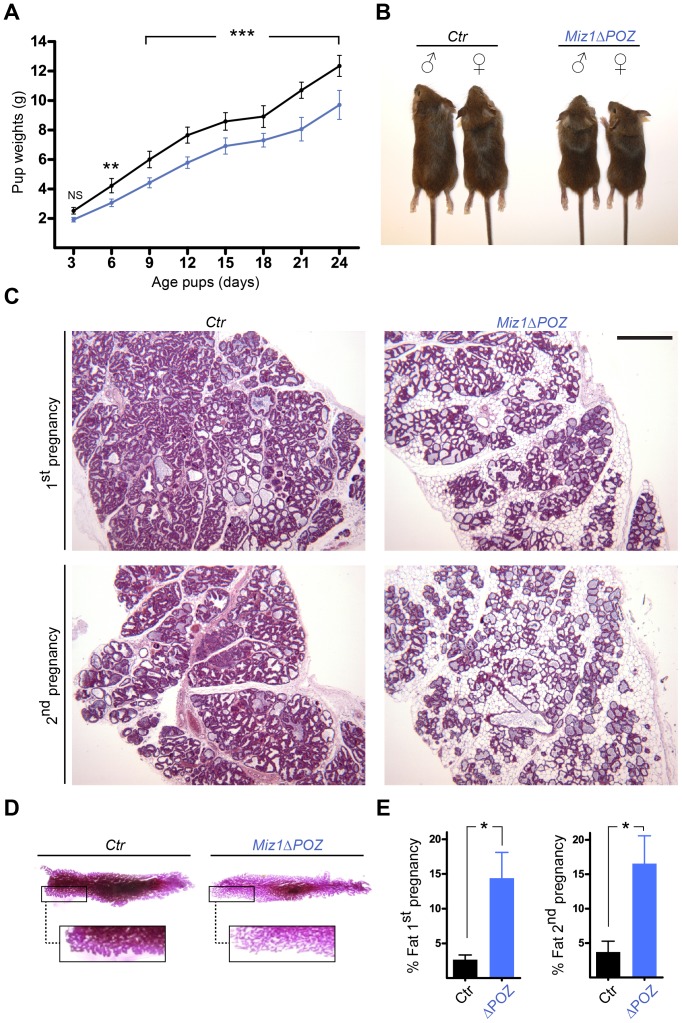
Female mice with a *Miz1*Δ*POZ* mammary gland exhibit a lactation defect. (**A**) Time course of the body weight of the offspring from control (black, n = 6) and *Miz1*Δ*POZ* mothers (blue, n = 6). The number of pups per mother was set to 6 at birth. (**B**) Size differences in 24-day-old pups did not depend on their gender. (**C**) Mammary glands from mothers of lactation day 6 were investigated by histology with H & E sections and glands from mothers of lactation day 1 were analysed in whole mounts (**D**). Morphometric analysis of the adipose tissue content from H & E sections (Lactation day 6) are shown in (**E**). Note that the difference in the ratio of glandular to adipose tissue is similar during the first and second pregnancies. Scale bar in C: 500 µm.

### Miz1 Mutant Mothers Feature a Lactation Defect

To test whether female mice with a homozygous deletion of the Miz1 POZ domain (hereafter referred to as *Miz1*Δ*POZ*) in luminal mammary gland epithelial cells have a lactation defect, we monitored the weight of newborn pups [43]. The number of pups per mother was set to six at birth and the offspring from six mothers per genotype were analysed (n = 6). On day three postpartum (3 pp) no significant difference in the body weight of the pups was observed (p>0.05), but at day 6 pp the weight of the pups fostered by *Miz1*Δ*POZ* mothers was significantly reduced (p<0.01) and this difference increased until day 24 pp (p<0.001; Fig. 2A and B). Histology of mammary glands from day 6 of lactation revealed a higher proportion of adipose tissue in *Miz1*Δ*POZ* mothers compared to control animals (Fig. 2C and Fig. S3). This was also observed during a second pregnancy and was further confirmed by whole mount preparations, which exhibited a less dense package of alveoli in *Miz1*Δ*POZ* animals (Fig. 2C and D). Using a morphometric approach we measured two to four percent of adipose tissue in wildtype mothers on lactation day 6 versus about 15% in Miz1 mutant mothers (1^st^ pregnancy: p = 0.021/n = 4; 2^nd^ pregnancy: p = 0.041/n = 3) (Fig. 2E). In contrast, histology of lactation day 10 control mammary glands did not differ from *Miz1*Δ*POZ* at this time point (Fig. S3), indicating that deletion of the Miz1 POZ domain caused a delay in mammary gland development during late pregnancy and early lactation.

**Figure 3:**
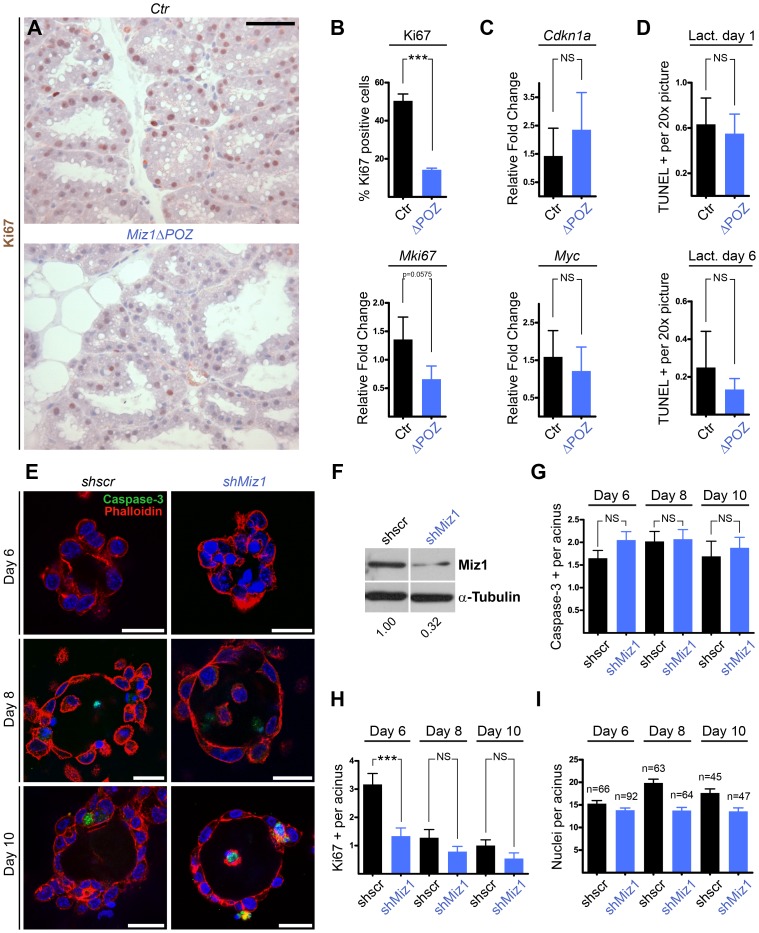
Miz1 function on proliferation and apoptosis of mammary gland epithelial cells *in vitro* and *in vivo*. (**A**) Ki67 immunostaining in control and *Miz1*Δ*POZ* lactation day 6 mammary glands. (**B**) Ki67 labeling index (up) and quantitative RT-PCR (down) obtained from lactation day 6 samples. (**C**) The expression of potential proliferation regulators like *Cdkn1a* and *Myc* was measured by qRT-PCR. (**D**) The TUNEL assay was performed on tissue from lactation day 1 and lactation day 6, respectively. Quantifications obtained from 20x pictures are shown (at least n = 3 per genotype and time-point). (**E**) Representative confocal microscopy pictures of acinar structures formed 6, 8 and 10 days after seeding *shscr* and *shMiz1* transfected HC11 cells onto Cultrex-coated coverslips. Nuclei were stained with Hoechst (blue), actin filaments with Phalloidin-TRITC (red) and apoptotic cells by cleaved Caspase-3 immunostaining (green). (**F**) Western Blot showing the knock-down of Miz1 in *shMiz1* HC11 cells. Numbers indicated are fold changes of band intensities obtained by densitometry (see Materials and Methods). Acinar cell apoptosis and proliferation were quantified by analysis of cleaved caspase-3 (**G**) and Ki67 (**H**) positivity in double immunofluorescence confocal microscopy pictures. (**I**) Quantification of the number of nuclei per acinus in *shscr* and *shMiz1* transfected HC11 cells. Data from two independent experiments were merged and the total number of acini analysed is indicated. See Materials and Methods for experimental details. Scale Bar in A: 50 µm; E: 25 µm.

Next, we analysed the proliferation of mammary gland epithelial cells by immunostaining for Ki67 on lactation day 6. There was a reduction in the number of Ki67 positive cells in *Miz1*Δ*POZ* animals (Fig. 3A). At this time point the Ki67 labeling index was almost three times lower in *Miz*Δ*POZ* mice compared with control animals (Fig. 3B/up). The Ki67 mRNA level was also reduced in *Miz1*Δ*POZ* animals, but the decrease was not statistically significant (p = 0.0575; Fig. 3B/down). The expression of *Cdkn1a* and *Myc* was not significantly changed (Fig. 3C). This suggests that the delay in mammary gland development during early lactation is caused by a reduced proliferation of the epithelial cells largely independent of Myc and p21^cip1^. To further elucidate whether the transient reduction of the mammary gland tissue depends on a higher rate of apoptosis we performed TUNEL assays on day 1 and 6 of lactation. At both time points, TUNEL positive cells were rare and their frequency was not different between wildtype and *Miz1*Δ*POZ* animals (Fig. 3D). These data were confirmed by immunohistochemical staining of cleaved caspase-3 (data not shown).

To confirm the role of Miz1 on the proliferation of mammary gland epithelial cells and to test for its impact on alveologenesis in a cell-autonomous manner, we used the mouse mammary gland derived cell line HC11 [44]. We generated stably transfected HC11 cells with expression vectors encoding either a scrambled short hairpin RNA (*shscr*) or a Miz1 specific short hairpin RNA (*shMiz1*) to knockdown Miz1 (Fig. 3F). We used these cells in an *in vitro* acini morphogenesis assay [36]–[38]. While apoptosis, measured as cleaved caspase-3 positive cells, was not affected by Miz1 depletion (Fig. 3G), Ki67 positive cells were reduced significantly on day 6 after seeding (Fig. 3H), the number of nuclei per acinus was decreased on days 8 and 10 (Fig. 3I) and the establishment of a lumen was delayed (data not shown). Taken together, our data provide evidence that mammary gland epithelial cells did not orderly proliferate without functional Miz1, while apoptosis was unaffected.

### *Miz1*Δ*POZ* Animals Show Altered Differentiation of Mammary Epithelial Cells

**Figure 4:**
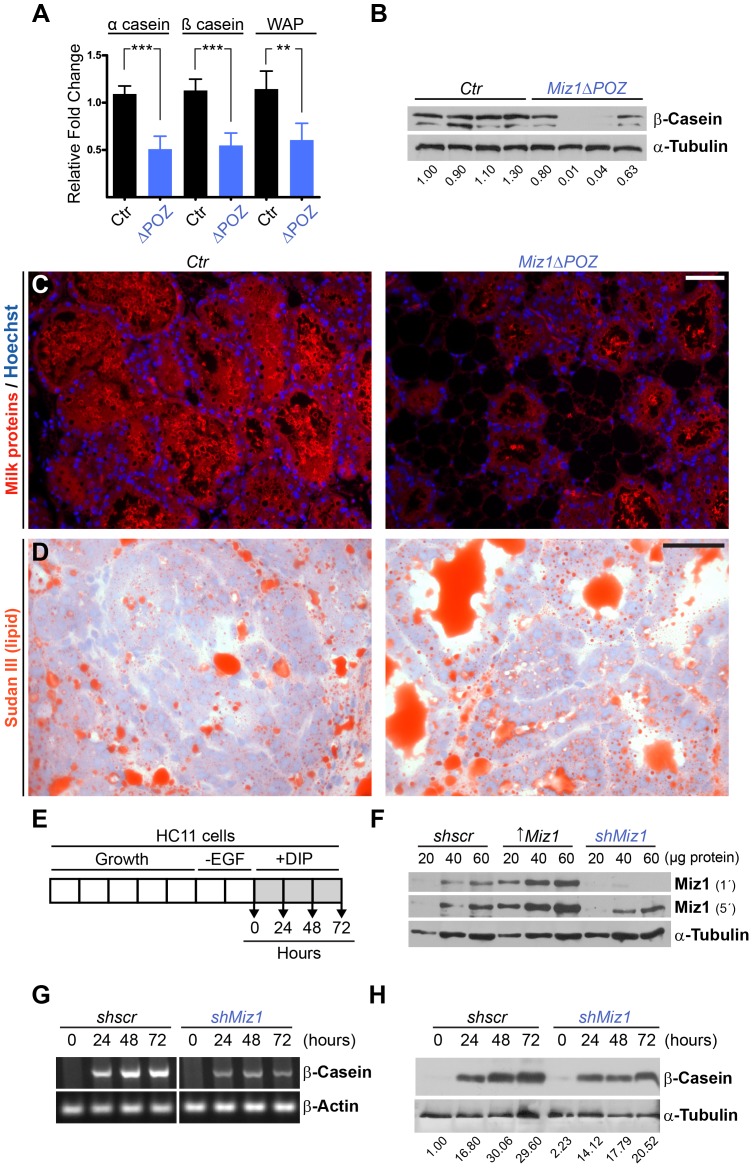
Differentiation of mammary gland epithelial cells in control (*Ctr*) and *Miz1*Δ*POZ* tissue and in HC11 cells with reduced levels of Miz1. (**A**) Gene expression of the milk proteins α-casein, ß-casein and whey acidic protein (Wap), measured by quantitative RT-PCR, in control and *Miz1*Δ*POZ* mammary gland tissue (ΔPOZ). (**B**) Immunoblot analysis for ß-casein from mice with the indicated phenotype. Each lane represents an individual animal. (**C**) Immunostaining with an antibody against milk proteins in the acini from control and *Miz1*Δ*POZ* mammary glands. (**D**) Sudan III staining was performed on cryosections from lactating mammary glands of *Ctr* and *MizΔPOZ* mice (n = 4 per genotype). Data from A to D was obtained from lactation day 6 samples. (**E**) Time course of growth and differentiation as performed in the experiments with HC11 cells. EGF: epidermal growth factor; DIP: differentiation media containing dexamethasone, insulin and prolactin. Time points indicated correlate to the time points in (**G**) and (**H**). (**F**) HC11 cells were stably transfected with scrambled short hairpin (sh) RNA (shscr), a Miz1 shRNA and a vector expressing Miz1 as a positive control. The film was exposed 1 and 5 minutes, respectively. (**G**) PCR and (**H**) Western blots revealed a down-regulation of ß-casein when Miz1 concentration is decreased. Numbers indicated in (**B**) and (**H**) are fold changes of band intensities obtained by densitometry (see Materials and Methods). Scale Bar in C: 50 µm; D: 100 µm.

Although a reduction in mammary gland tissue of *Miz1*Δ*POZ* mothers is no longer visible on lactation day 10, the reduced pup weight is not rescued but the difference between wildtype and Miz1 mutant animals is even increasing. In order to identify genes potentially regulated by Miz1 which could explain the observed phenotype and to assess the relative expression of milk protein genes, a genome-wide cDNA microarray was performed using samples from control and *Miz1*Δ*POZ* animals obtained at day 6 of lactation (n = 4 for each genotype; Fig. S4A and B). Here, 35% of all regulated genes were up-regulated in *Miz1*Δ*POZ* animals, while in approximately 65% the gene expression was down-regulated, indicating that Miz1 is predominantly transactivating genes or enhancing gene activity indirectly. Different casein genes (*Csn1s1, Csn1s2a, Csn1s2b, Csn2, Csn3*) and the whey acidic protein gene (*Wap*) were up to 2.5fold down-regulated in *Miz1*Δ*POZ* animals (Fig. S4B). This could be confirmed by quantitative RT-PCR which revealed a twofold down-regulation of the genes encoding α-casein (p<0.001), ß-casein (p<0.001) and whey acidic protein (p<0.01) (Fig. 4A). Western blots using an antibody against ß-casein exhibited a reduced content of this milk protein in mammary gland tissue from *Miz1*Δ*POZ* animals (Fig. 4B). In addition, less milk protein was present in the alveoli from the mutant animals compared with control animals (Fig. 4C) on sections of lactation day 6 mammary glands, stained with an antibody against mouse milk proteins [45]. Furthermore, fat droplets in the lumina of the alveoli from *Miz1*Δ*POZ* mothers coalesced to larger aggregates which were not observed in control animals to this extent (Fig. 4D). A similar phenotype has been reported previously [46] where the lipid droplet defect was attributed to an impaired calcium transport caused by a deregulation of calcium transporter genes including *Camk2b* and *Ano4* (down-regulated), and *Clca1* and *Clca2* (upregulated). Of note, gene expression of these four proteins was deregulated in the same manner in our cDNA microarray analysis (*Clca1*: 4.6-fold up; *Clca2*: 5.4-fold up; *Camk2b*: 3.8-fold down; *Ano4*: 1.6-fold down) and this was further confirmed by quantitative RT-PCR (Fig. S4C). In addition, a group of genes, usually expressed during an immune response, was up-regulated (Fig. S4D). Similar results were recently obtained in the lung, showing Miz1 as a suppressor of inflammation [47]–[49].

To test whether the altered milk protein expression can also be observed on a cellular basis, we again used the mouse mammary gland derived cell line HC11, which can undergo a limited functional differentiation under appropriate hormonal stimulation [35] (Fig. 4E). Experiments were performed with cells stably transfected with a *shscr* or an *shMiz1* expression vector [34] to knock down Miz1 (Fig. 4F). *shscr* cells expressed *Csn2* (encoding ß-casein) mRNA in a time dependent manner after stimulating the cells with prolactin (Fig. 4G). In contrast, *Csn2* expression was greatly reduced in *shMiz1* cells (Fig. 4G) and the lower expression of *Csn2* mRNA resulted in a lower amount of ß-casein protein (Fig. 4H). Taken together, a reduction in the levels of a functional Miz1 protein led to a lower expression and synthesis of milk proteins in luminal mammary gland cells and thus to a decreased differentiation of mammary gland tissue.

### Deficient STAT5 Function in the Mammary Gland of Miz1 Mutant Mice

**Figure 5:**
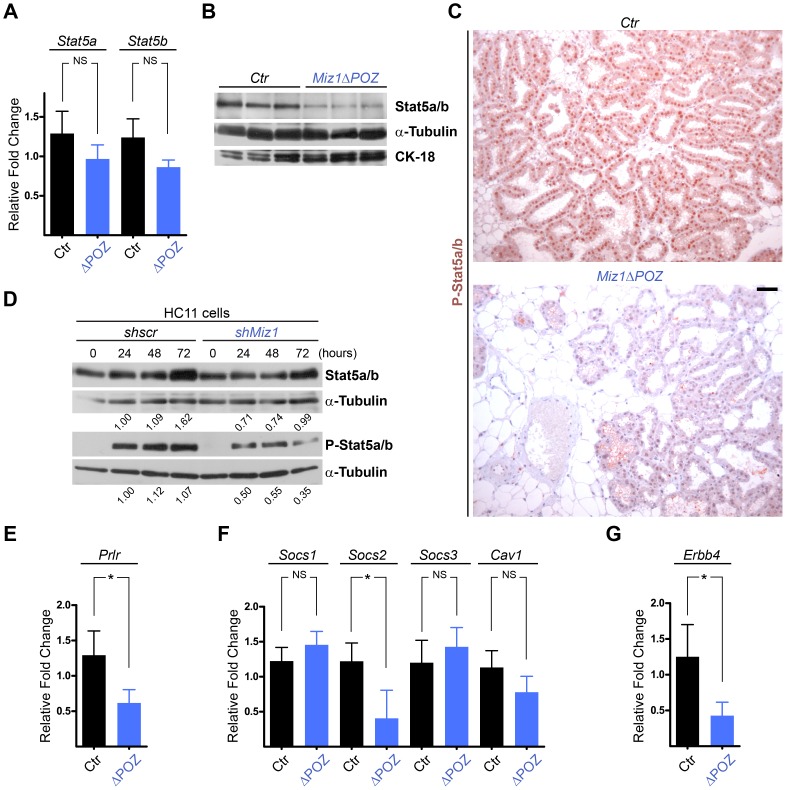
Stat5 phosphorylation is reduced in *Miz1*Δ*POZ* mammary glands and in *shMiz1* HC11 cells. (**A**) Analysis of Stat5a/b mRNA expression in *Miz1*Δ*POZ* (ΔPOZ) and control mammary gland tissue (*Ctr*). (**B**) Immunoblot analysis of Stat5 in wildtype and *Miz1*Δ*POZ* mammary glands. (**C**) Immunohistochemical staining of pStat5 in the mammary gland tissue from wildtype and *Miz1*Δ*POZ* animals. (**D**) Western blots from HC11 cells stably transfected with a scrambled short hairpin (sh) RNA (shscr) or a Miz1 shRNA (see also Fig. 4**E** and **F**). Numbers indicated are fold changes of band intensities obtained by densitometry (see Materials and Methods). Quantitative RT-PCR for the prolactin receptor (**E**; *Prlr*), the Supressors of cytokine signalling (*Socs*) 1, 2 and 3 and caveolin-1 (**F**; *Cav1*), and for *ErbB4* (**G**). Lactation day 6 samples were used in all *in vivo* experiments. Scale bar in C: 50 µm.

Signal Transducer and Activator of Transcription (Stat) 5a and 5b have shown to be the key signalling molecules in proliferation, differentiation and survival of mammary gland epithelial cells [9], [11], [50]. We measured the expression of *Stat5a/b* by quantitative RT–PCR and observed a slight but statistically not significant decrease of the Stat5a/b mRNA in *Miz1*Δ*POZ* mice (Fig. 5A). However, in Western blots the Stat5 protein was less expressed in *Miz1*Δ*POZ* mammary glands compared to wildtype animals (Fig. 5B). Stat5 is activated by phosphorylation either by Jak2, associated with cytokine or hormone receptors like the prolactin receptor, or directly by ErbB4 [9]. On lactation day 6, phosphorylated Stat5 (pStat5) was decreased, both in regard to the number of nuclei stained, as well as to the staining intensity, using immunohistochemical stainings of mammary gland sections from control and *Miz1*Δ*POZ* animals (Fig. 5C). To test again whether this can be confirmed in a cell-autonomous model, we knocked-down Miz1 in HC11 cells and analysed Stat5 expression and phosphorylation at different time points after addition of prolactin. Although the amount of Stat5 was not as obviously reduced as *in vivo*, phosphorylation was clearly decreased (Fig. 5D). Taken together, these data show that the Stat5 amount and phosphorylation were diminished *in vivo* and *in vitro* when functional Miz1 was absent.

As shown in Fig. 5D, prolactin is a strong stimulator of Stat5 phophorylation in HC11 cells and this has also been described for the mammary gland in the literature [50]. First, we tested whether the expression of the prolactin receptor is altered in *Miz1*Δ*POZ* animals and found that its expression is about 2-fold reduced compared to control mice (Fig. 5E and Fig. S4B). Next, we analysed the expression of *Socs1* (encoding Suppressor of cytokine signalling 1/Socs1) and *Cav1* (encoding caveolin-1), which have been shown to down-regulate the Jak2 kinase activity [50]. The expression of both genes was not significantly altered and this was also true for *Socs3* (Fig. 5F and Fig. S4B). Interestingly, the expression of *Socs2*, a direct target gene of Stat5 [51], was 2–3fold down-regulated (Fig. 5F and Fig. S4B), confirming further an alleviated Stat5 signalling pathway. In addition to the prolactin receptor/Jak2 mediated activation of Stat5, ErbB4 has been identified as an obligate direct mediator of Stat5 phosphorylation and nuclear translocation in the mammary gland [52], [53]. As shown in Fig. 5G, the expression of the ErbB4 gene was significantly reduced in mammary glands from *Miz1*Δ*POZ* animals (see also Fig. S4B).

### ChIP-Seq Reveals Miz1 as a Regulator of Vesicular Transport Gene Expression

**Figure 6:**
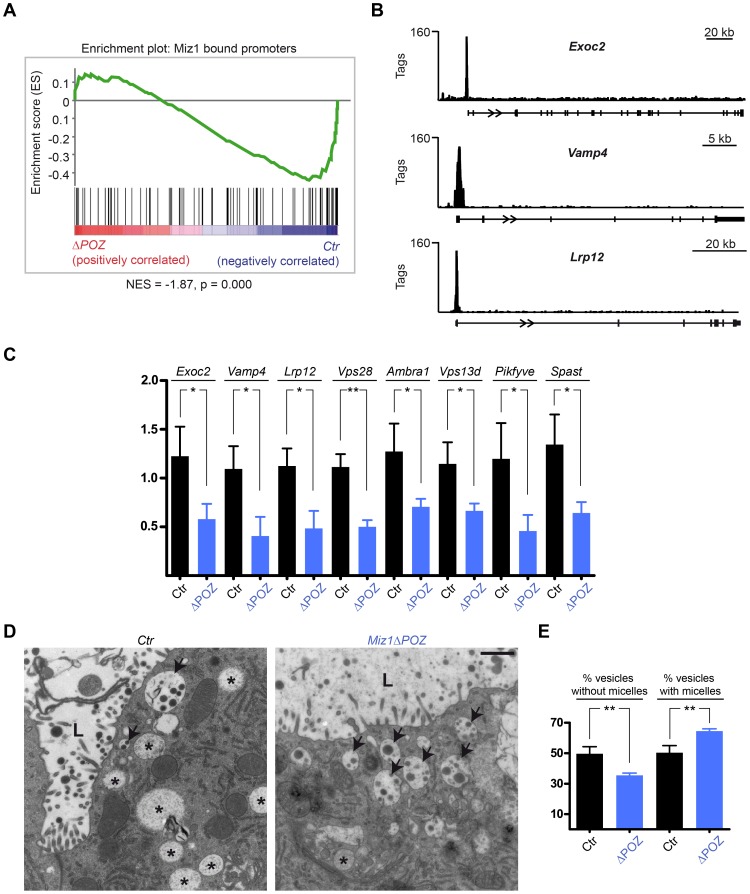
Genes related to vesicular transport processes are bound by Miz1 and down-regulated in *Miz1*Δ*POZ* mammary glands. (**A**) GSEA analysis comparing the gene expression of wildtype versus *Miz1*Δ*POZ* mammary glands. The 100 Miz1 target promoters with the highest tag number were used as the gene set in this analysis. (**B**) Browser pictures of Miz1 ChIP-Seq profiles at the Miz1 target genes *Exoc2*, *Vamp4* and *Lrp12.* (**C**) Quantitative RT-PCRs testing the expression of genes indicated in Table 1. (**D**) Electron microscopy showing vesicles with (arrows) and without (asterisks) casein micelles in tissue from control and *Miz1*Δ*POZ* animals. (**E**) Percentage of the two vacuole types in mammary gland epithelial cells of control and *Miz1*Δ*POZ* animals from lactation day 10. Data obtained from 4 animals per genotype. Total number of vacuoles counted: ctr, 309–379; ΔPOZ, 339–404. Scale bar in D: 1 µm.

**Figure 7:**
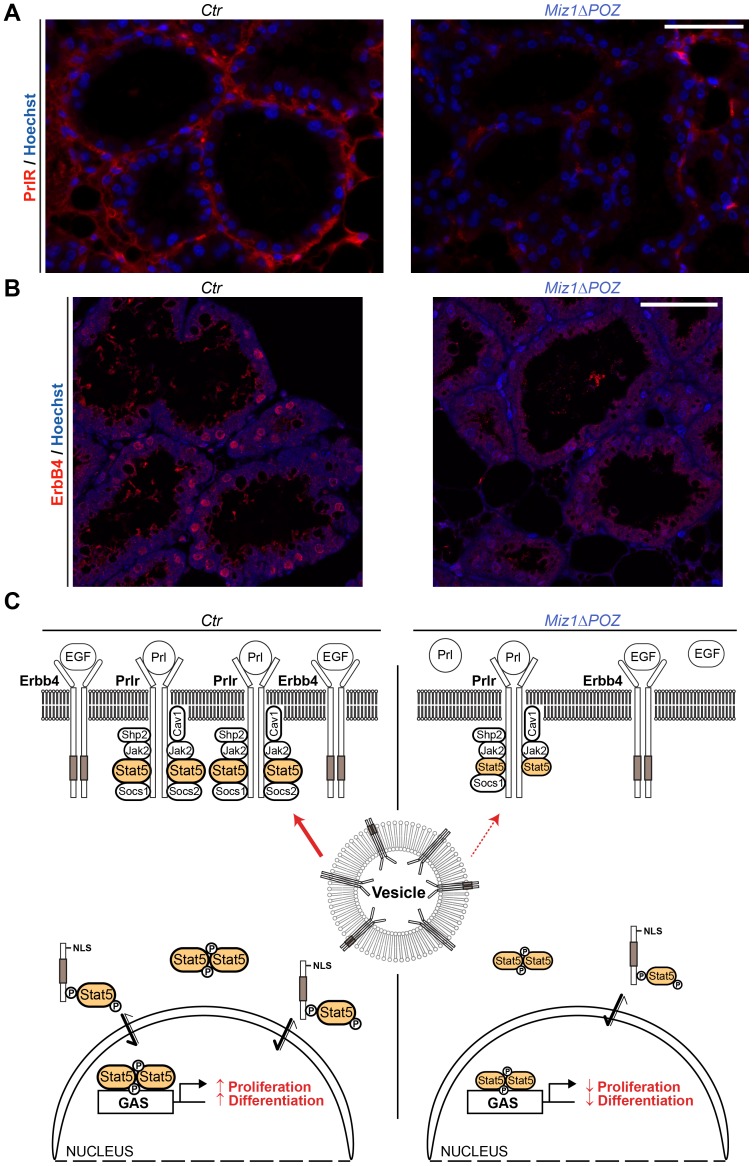
Protein expression and localization of Prlr and ErbB4. PrlR (**A**) and ErbB4 (**B**) immunofluorescence from control and *Miz1*Δ*POZ* mammary gland tissue (lactation day 6). (**C**) Hypothetical model about the function of Miz1 in the mouse lactating mammary gland. Vesicular transport processes are impaired in *Miz1*Δ*POZ* mice due to a decreased gene expression of Miz1 target genes which are involved in the vesicular transport. This causes a reduction of the PrlR and ErbB4 exposure to the plasma membrane, hampering the autoamplifying expression of PrlR. Reduction of PrlR and ErbB4 expression and their diminished availability at the cell surface leads to a decreased amount of phosphorylated Stat5, which is the key regulator during lactation. In consequence, reduced levels of phosphorylated Stat5 dimers (represented by smaller symbol size) cannot adequately activate the transcription of proliferation and differentiation genes in *Miz1ΔPOZ* glands [50], [53]. Scale bars in A and B: 50 µm.

In order to gain insight into the mechanism underlying the observed phenotype, we performed Miz1 ChIP-Seq experiments using the mammary epithelial cells MDA-MB231 [54]. We identified 830 promoters bound by Miz1. To analyse how Miz1 regulates these target genes during lactation, we created a gene set with the 100 most strongly Miz1 bound genes and correlated this list with the gene expression data from our cDNA microarray experiments performed on day 6 of lactation. This gene set enrichment analysis (GSEA) showed that a majority of Miz1 target genes are down-regulated in *Miz1*Δ*POZ* animals (Fig. 6A and B). Table 1 summarizes all down-regulated genes which had at least 200 binding tags in the ChIP-Seq experiment. 12 out of 23 identified genes encode for proteins related to vesicular transport processes and evaluation by quantitative RT-PCR confirmed the expression data from the microarray analysis (Fig. 6C). Interestingly, ultrastructural analysis of the secretory vacuoles (Fig. 6D) revealed a higher percentage of casein micelle containing vacuoles (p<0.0011) on lactation day 10 in *Miz1*Δ*POZ* animals (Fig. 6E), combined with a higher, but statistically not significant, mean number of micelles per vacuole (3.14±0.56 in control and 4.42±0.40 in *Miz1*Δ*POZ* animals; p = 0.1127), pointing to a derangement of the development, maturing or transport of the secretory vesicles. Further, in the ChIP-Seq experiment Miz1 did neither bind *Prlr* nor *Erbb4*, showing that both genes are not direct target genes of Miz1. With an immunohistochemical approach, prolactin receptor was hardly detectable in the plasma membrane of the epithelial cells from *Miz1*Δ*POZ* lactating mammary glands (Fig. 7A), allowing the hypothesis that not only a reduced expression but also an impaired intracellular vesicular transport attenuated the prolactin receptor signalling. In line with this observation was a reduction of ErbB4 in the nuclei of the epithelial cells (Fig. 7B), since this receptor has to be transported to the plasma membrane before the cleaved cytosolic domain can target the nucleus. Taken together our data are compatible with the notion that a deletion of the Miz1 POZ domain has a pleiotropic effect on the intracellular vesicular transport machinery. Subsequently, signalling processes like Stat5 phosphorylation, which depend on correctly transported and targeted plasma membrane proteins, like the prolactin receptor or ErbB4, are impaired.

**Table 1:** GSEA analysis.

gene symbol	binding [tags]	regulation ΔPOZ/ctr[log2FC]	process/function
*Gphn*	1355	−0,64397	protein localization,synaptic structure
***Exoc2/Sec5***	**1173**	−**0.78775**	**membrane Trafficking, translocation of GLUT4 to the plasma Membrane**
*Mrps23*	1158	−0,41446	structural ribosomal protein
***Vamp4***	**1105**	−**0.98223**	**SNARE interactions in vesicular transport**
***Tbc1d14***	**1029**	−**0.40866**	**regulation of autophagic vacuole assembly**
***Lrp12***	**977**	−**0.74450**	**endocytosis**
*Heatr5a*	949	−0,55225	unknown
***Vps28***	**899**	−**0.87396**	**endocytosis**
*Wdr13*	893	−0,42085	unknown
***Dctn6***	**873**	−**0.93368**	**dynein dependent vesicular transport**
*Tpk1*	863	−0.42197	thiamine metabolism
***Ambra1***	**841**	−**0.48620**	**regulation of autophagic vacuole assembly**
*Pdcd5/*TFAR19	795	−1.00497	apoptosis
***Vps13d***	**736**	−**0.23141**	**Golgi localisation**
***Pikfyve***	**729**	−**0.48238**	**phosphatidylinositol metabolic process, endocytosis**
***Snx18/*** **Snag1**	**724**	−**0.56591**	**endosomal transport**
*Tbxas1*	702	−0.66024	oxidation-reduction process,iron/heme metabolism
*Tcea1*	645	−0.83292	regulation of DNA-dependent transcription
*Tmbim4*	645	−0,49351	apoptosis
*Nrip1*	445	−0,54765	mammary gland development,energy metabolism
***Spast***	**428**	−**0.47523**	**microtubule dependent vesicular transport**
***Dync2h1***	**374**	−**0.56335**	**dynein dependent vesicular transport**
***Inpp5A***	**351**	−**0.43089**	**phosphatidylinositol metabolic process, endocytosis**

ChIP-Seq data from MDA-MB231 cells were combined with microarray expression data obtained from control and *Miz1*Δ*POZ* mammary gland tissue at lactation day 6. Listed are genes which show a strong Miz1 binding (>200 binding tags) and a down-regulation in *Miz1*Δ*POZ* animals. Genes which are related to vesicular transport processes are highlighted in bold.

## Discussion

Deletion of the Miz1 POZ domain in mammary gland epithelial cells rendered a functionally mutated Miz1 protein which caused a lactogenic defect in the first and second pregnancies. This lactation defect was characterized by a diminished proliferation and differentiation of the glandular cells which caused a delayed development of the mammary gland during lactation and resulted in malnutrition of the pups. In the normal mammary gland, Miz1 levels were low at late pregnancy (P18.5) but increased dramatically at the transition from pregnancy to lactation (L1), where it remained high until involution (I2). In line with this notion, phenotypical differences of the mammary gland between control and *Miz1*Δ*POZ* animals, like a transient reduction of the glandular tissue, a decrease of milk protein expression and an extracellular coalescence of lipid droplets, became visible not before lactation, although the Cre recombinase under the control of the Wap promoter was expressed already at day 14.5 of pregnancy [6]. The sudden increase and decrease of Miz1 protein levels at the beginning and the end of lactation, respectively, supports the data provided, which show that Miz1 has an important function in initiating and maintaining the lactation state of the mammary gland.

Miz1 was originally found as a Myc binding protein [15] and it was shown that the binding of Myc/Max complexes to Miz1 at the initiation region of a promoter represses gene expression [21], [30]. This has been well documented for *Cdkn1a* and *Cdkn2b*, encoding the cyclin dependent kinase inhibitors p21^cip1^ and p15^ink4b^, respectively. In skin, it was shown that a functional mutation of Miz1 in keratinocytes of the basal epidermal cell layer reveals an increase of p21^cip1^ as a result of a missing Miz1/Myc repressing complex. This leads to a reduced proliferation, a maintained or even increased differentiation and an alleviated development and growth of induced skin papilloma, all of which can be rescued on a p21^cip1^ null background [24]. However, in our ChIP-Seq analysis in the mammary gland epithelial cell line MDA-MB231 neither *Cdkn1a* nor *Cdkn2b* were present under the 830 genes bound by Miz1, indicating that it does not regulate these genes in this cell type. This is in agreement with the observation that the expression of *Cdkn1a* was slightly but statistically not significantly increased in the mammary gland from *Miz1*Δ*POZ* animals, making it unlikely that the reduced proliferation observed is primarily caused by upregulated p21^cip1^. In contrast to Miz1 expression, the Myc protein level is high in early pregnancy, peaking at day 6, remaining elevated until day 12.5 and declining to basal levels prior to day 18.5 [7]. This suggests that Myc plays a pivotal role in mammary gland development during early pregnancy, most likely by stimulating proliferation to expand the glandular tissue. Myc expression during lactation could not be detected (Fig. S1E). Moreover, about two thirds of all regulated genes in *Miz1*Δ*POZ* lactating mammary gland tissue were down-regulated in the array (Fig. S4A). This indicates that Miz1 is acting more as a transactivator and not as a repressor in conjunction with Myc, most likely because there are less repressive complexes due to low Myc expression. However, when the Myc gene was deleted during mid to late pregnancy in a conditional mouse model using *Wap-Cre*, a lactation phenotype was also observed [6]. Phenotypically, the authors describe a delayed proliferation and differentiation, impaired translation of milk proteins and a reduction of mammary gland precursor cells. Whether some of these observations are linked to the absence of Myc/Miz1 repressive complexes remains to be elucidated. However, a significant upregulation of p21^cip1^, as observed in *Miz1*Δ*POZ* skin [24] and Myc-deficient mammary gland [6], was not observed in the *Miz1*Δ*POZ* mammary gland, suggesting that a relief of *Cdkn1a* repression by Myc/Miz1 is not the main reason for the reduction in proliferation. Moreover, keratinocyte differentiation was enhanced in *Miz1*Δ*POZ* skin in a p21^cip1^ dependent manner [24] while differentiation of *Miz1*Δ*POZ* luminal mammary gland cells was decreased.

The signal transducer and activator of transcription (Stat) 5, especially Stat5a, establishes a central signalling node for proliferation and differentiation of the luminal mammary gland epithelium, as well as for alveologenesis during pregnancy and lactation [50]. When Stat5 was conditionally deleted in late pregnancy using *Wap-Cre*, a similar reduction in mammary gland tissue was observed as in our animal model [12]. More sophisticated experiments revealed that the extent of glandular tissue that develops in late pregnancy and lactation depends on the Stat5 concentration in the luminal cells [11]. In parallel to the morphological phenotype, genes encoding milk proteins or proteins being involved in the regulation of luminal cell proliferation and differentiation were gradually down-regulated with different Stat5 dosages [11]. In line with these observations is the mammary gland specific knockout of Ski novel protein (SnoN) [55]. Deletion of SnoN, which stabilizes the Stat5 protein, reduced Stat5 concentrations in luminal mammary epithelial cells and induced a lactogenic defect resembling the phenotype seen in Stat5 knockouts or in the *Miz1*Δ*POZ* mammary gland. *Miz1*Δ*POZ* animals also exhibited a reduced amount of Stat5 compared to control tissue when analysed by Western blots. Since we used the mammary gland epithelial cytokeratin-18 as a loading control, the difference of Stat5 concentrations cannot be attributed to a different ratio between adipose and glandular tissue. Whether the subtle decrease of Stat5a/b gene expression is sufficient to explain the reduced Stat5 protein in mutant glands, or if the deletion of the Miz1 POZ domain inhibits Stat5 translation or promotes Stat5 degradation, remains to be elucidated. Further, immunohistochemistry revealed a clear decrease in phosphorylated Stat5. Taken together, we conclude that the reduced proliferation and differentiation in the mammary gland of *Miz1*Δ*POZ* animals during lactation depends on a lower amount of phosphorylated Stat5, brought on by a reduced Stat5 amount and phosphorylation.

Stat5 is phosphorylated by a variety of cytokine receptors depending on the cell type [9]. Cytokine receptors recruit Jak2 which finally phosphorylates Stat5 [56]. The Jak2-Stat5 pathway is altered in lymphocytes when functional Miz1 is missing, mainly by the up-regulation of the Suppressor of cytokine signalling 1 (Socs1) in response to interleukin-7 stimulation [25]. In the mammary gland both, *Socs1* and *Socs2*, are target genes of activated Stat5 [50], but we observed only a lower *Socs2* expression in *Miz1*Δ*POZ* animals, while *Socs1* was not altered and, in particular, not up-regulated like in mutant B-cells [25]. Interestingly, Socs genes do not occur in the Miz1 binding list from our ChIP-Seq data, indicating that they are not Miz1 target genes in mammary gland cells, in contrast to B-cells, where *Socs1* expression is directly regulated by Miz1 [25].

In mammary gland cells the Jak2-Stat5 pathway is activated by the prolactin receptor [57]. The expression of this receptor was significantly down-regulated in *Miz1*Δ*POZ* animals, providing a possible explanation for the reduced pStat5. In line with this notion is the observation that heterozygous knockout of the prolactin receptor in the mouse mammary gland leads to a similar phenotype as seen in the *Miz1*Δ*POZ* animals [51]. The knockout phenotype was rescued on a Socs2 null background indicating that half dosage of the prolactin receptor is sufficient when the Jak2 inhibitor Socs2 is absent. However, although Socs2 was down-regulated in the mammary gland of *Miz1*Δ*POZ* mice, Stat5 was less phosphorylated suggesting that either the amount of Stat5 itself was low or the concentration of the prolactin receptor was not in favor of a more efficient Stat5 phosphorylation. In addition, it has been shown that ErbB4 also phosphorylates Stat5 in a Jak2 independent manner in the mammary gland epithelium [52] and promotes the nuclear translocation of pStat5 [53]. Of note, the mRNA of this protein was also decreased in *Miz1*Δ*POZ* animals providing an additional explanation for the observed decrease of pStat5.

Our data from ChIP-Seq experiments suggest that *PrlR* and *ErbB4* are not directly regulated by Miz1. In contrast, about 50% of genes which bind Miz1 and are down-regulated in the mammary gland from *Miz1*Δ*POZ* animals are related to vesicular transport processes indicating that Miz1 influences multiple functions related to secretion or intracellular protein targeting including the transport of plasma membrane proteins to their final destination. This hypothesis is compatible with the observation that in *Miz1*Δ*POZ* animals 1) the composition of the secretory vesicles is altered, 2) ErbB4 concentration in the nucleus is reduced and 3) the PrlR is not properly located in the plasma membrane. Signalling by the PrlR depends not only on a correct localization at the plasma membrane [58], but is also modified by internalization via clathrin dependent or independent endocytosis [59], another branch of vesicular transport. Interestingly, it has been shown that transcriptional expression of *PrlR* is enhanced by Prl-induced PrlR signalling [60], [61], suggesting a link between the observed reduction of *PrlR* to an impaired vesicular transport.

*In vivo* studies during the last years revealed that Miz1 has pleiotropic functions in different tissues like skin [23], [24], B- and T-cells [25], [26] or cerebellum [62]. Interestingly, the absence of functional Miz1 led to attenuated tumorigenesis in mouse skin [24] and in a murine lymphoma model [63], either by up regulation of *Cdkn1a* expression or by induction of senescence via an autocrine TGFβ signalling loop, respectively. Together with the fact that Miz1 is a central player in mediating the repressive function of the protooncogene Myc in cancer [64], relevant roles of Miz1 in different kinds of tumors, also in the mammary gland, are likely. In addition, this first report about the physiological function of Miz1 in the mammary gland documents its importance for an adequate mammary cell proliferation and differentiation during lactation.

In conclusion, we propose a tentative model (Fig. 7C) where the lack of functional Miz1 causes a down-regulation of a gene set involved in vesicular transport, limiting a proper localization, degradation and recycling of plasma membrane proteins. This disrupts the Stat5 pathway due to impaired signalling via PrlR and ErbB4, reflected by an alleviated expression of Stat5 target genes like *Csn1s1, Csn1s2a, Csn1s2b, Csn2, Csn3, Wap* or *Socs2* as well as *PrlR* and *ErbB4*. This deficiency in Stat5 activation leads to a decrease in differentiation, together with a reduced proliferation, which cause the lactation phenotype in *Miz1*Δ*POZ* animals.

## Supporting Information

Figure S1 **c-Myc immunohistochemistry during murine mammary gland development.** c-Myc expression in inguinal mammary glands from *Ctr* animals was assessed by immunohistochemistry. The time-points analysed were: virgin gland at 45 days ppm (**A**; n = 3), pregnancy day 10.5 (**B**; n = 2), 14.5 (**C**; n = 2) and 18.5 (**D**; n = 3), lactation day 6 (**E**; n = 3) and involution day 2 (**F**; n = 3). Nuclear c-Myc staining was visible in virgin mammary ducts and in the forming alveoli during early pregnancy as described elsewhere by gene expression analysis [7], [65] and by immunohistochemistry of pregnancy day 6.5 animals [6]. During late pregnancy, a clear nuclear c-Myc staining was visible in the lymph node but not in the surrounding mammary alveoli (see arrows in D). c-Myc expression was not detectable during lactation [66] and was hardly discernible at involution day 2. Scale bars: 50 µm and 10 µm in the inset.
(TIF)

Figure S2 **Conditional deletion of the Miz1 POZ domain in luminal mammary epithelial cells.** (**A**) Schematic representation of the *Wap-Cre*-mediated recombination strategy used to delete the exons which code for the Miz1 POZ domain and the relative position of the primers to detect it [23]. (**B**) Time course of the appearance of the recombinant band performed on genomic DNA isolated from mammary glands at the indicated time-points. As described elsewhere [6], [28], Cre expression under the *Wap* promoter is weakly detectable already at pregnancy day 14.5 (B) and strong and sustained during late pregnancy and lactation as seen also by immunohistochemistry (**C, D**). Scale bars: 50 µm.
(TIF)

Figure S3 **Representative H&E stainings of** ***Ctr*** **and** ***Miz1ΔPOZ*** **animals.** In accordance with Miz1 expression levels during mammary development (Fig. 1), first pregnancy day 18.5 samples show similar alveolar density in *Ctr* and *MizΔPOZ* animals (**A, B**; n = 3 per genotype). The reduced alveologenesis phenotype only becomes apparent at lactation day 1 (**C, D;** at least n = 3 per genotype), obvious at lactation day 6 (**E, F** and Fig. 2; n = 8 per genotype) and partially rescued by lactation day 10 (**G, H**; n = 4 per genotype). Scale bar: 300 µm.
(TIF)

Figure S4 **Microarray analysis of lactation day 6 mammary glands.** (**A**) Number of regulated genes in *Miz1ΔPOZ* mammary glands using different Fold Change (FC) thresholds. Note the increased number of genes down-regulated in *Miz1ΔPOZ* animals. (**B** to **D**) Summary of microarray data, after normalization and filtering, showing the relative expression of different gene sets in *Miz1ΔPOZ* animals concerning (**B**) Stat5 target genes, Stat5 signalling and mammary epithelial cell proliferation, (**C**) calcium transport and (**D**) immune response. Fold changes were averaged when different values for the same gene were available. See Materials and Methods for experimental details.
(TIF)

Table S1 **Summary of all oligonucleotides used for PCR approaches.** Primers are provided in 5′→3′ direction. Quantitative PCR primers were designed using the Universal Probe Library Assay Design Center on-line tool (Roche Diagnostics, Mannheim, Germany).
(DOC)
