# Supplementary figures and images for: Miz1 Deficiency in the Mammary Gland Causes a Lactation Defect by Attenuated Stat5 Expression and Phosphorylation
Source: PLoS One. 2014 Feb 19;9(2):e89187. doi: 10.1371/journal.pone.0089187 (PMC3929623; doi:10.1371/journal.pone.0089187)

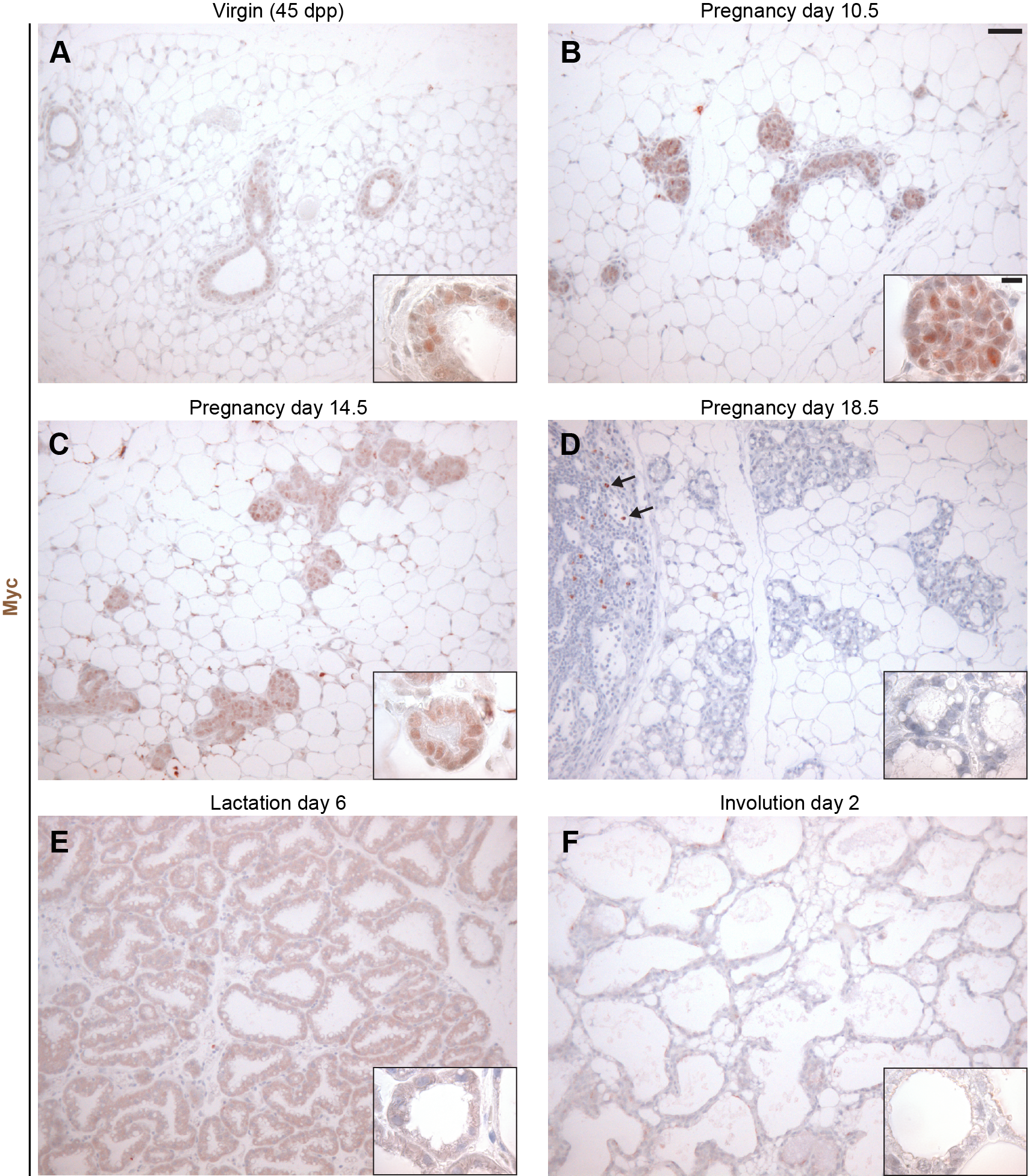

Supplement: Figure S1 — c-Myc immunohistochemistry during murine mammary gland development. c-Myc expression in inguinal mammary glands from Ctr animals was assessed by immunohistochemistry. The time-points analysed were: virgin gland at 45 days ppm (A; n = 3), pregnancy day 10.5 (B; n = 2), 14.5 (C; n = 2) and 18.5 (D; n = 3), lactation day 6 (E; n = 3) and involution day 2 (F; n = 3). Nuclear c-Myc staining was visible in virgin mammary ducts and in the forming alveoli during early pregnancy as described elsewhere by gene expression analysis [7], [65] and by immunohistochemistry of pregnancy day 6.5 animals [6]. During late pregnancy, a clear nuclear c-Myc staining was visible in the lymph node but not in the surrounding mammary alveoli (see arrows in D). c-Myc expression was not detectable during lactation [66] and was hardly discernible at involution day 2. Scale bars: 50 µm and 10 µm in the inset. (TIF) [file pone.0089187.s001.tif]

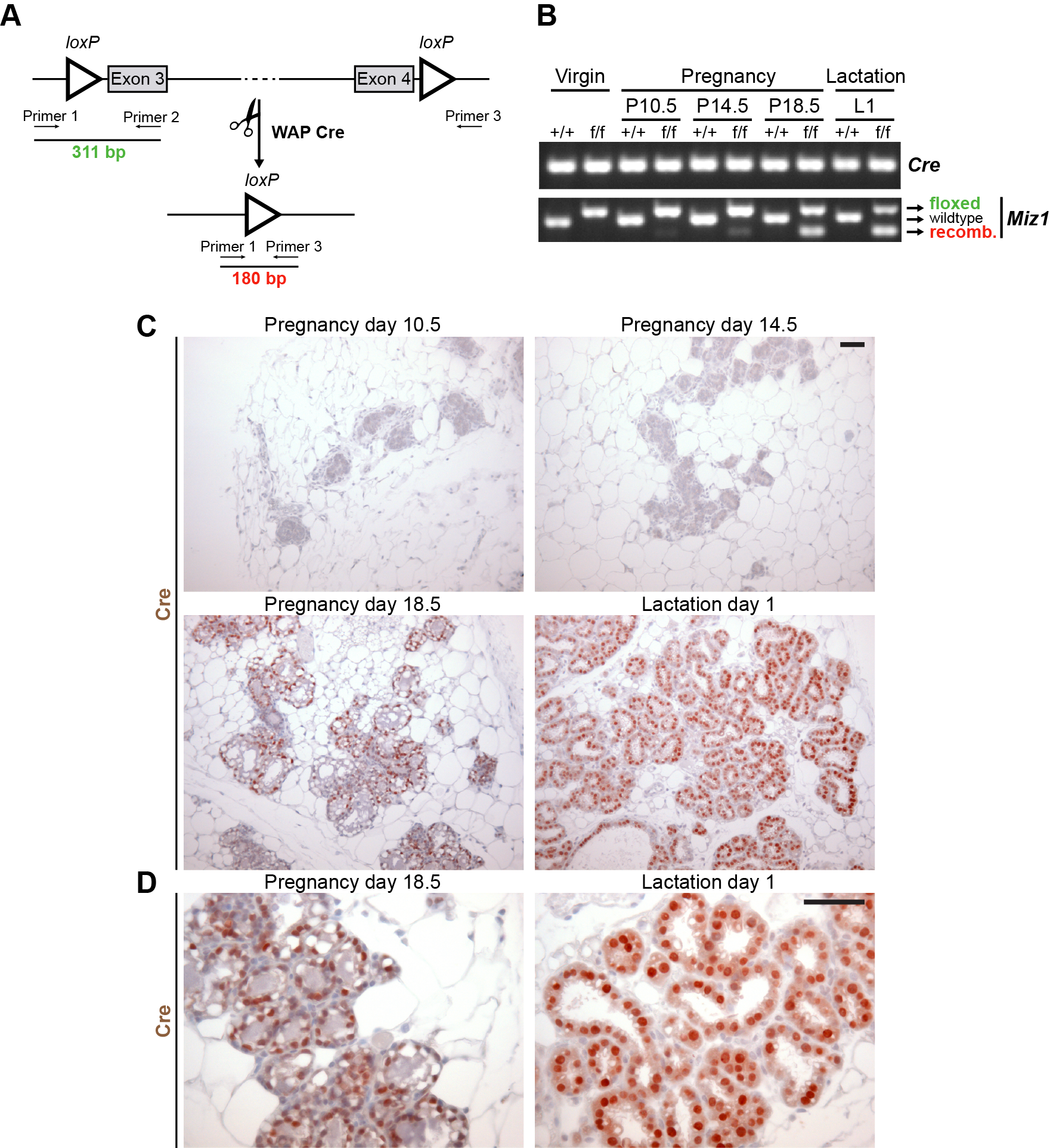

Supplement: Figure S2 — Conditional deletion of the Miz1 POZ domain in luminal mammary epithelial cells. (A) Schematic representation of the Wap-Cre-mediated recombination strategy used to delete the exons which code for the Miz1 POZ domain and the relative position of the primers to detect it [23]. (B) Time course of the appearance of the recombinant band performed on genomic DNA isolated from mammary glands at the indicated time-points. As described elsewhere [6], [28], Cre expression under the Wap promoter is weakly detectable already at pregnancy day 14.5 (B) and strong and sustained during late pregnancy and lactation as seen also by immunohistochemistry (C, D). Scale bars: 50 µm. (TIF) [file pone.0089187.s002.tif]

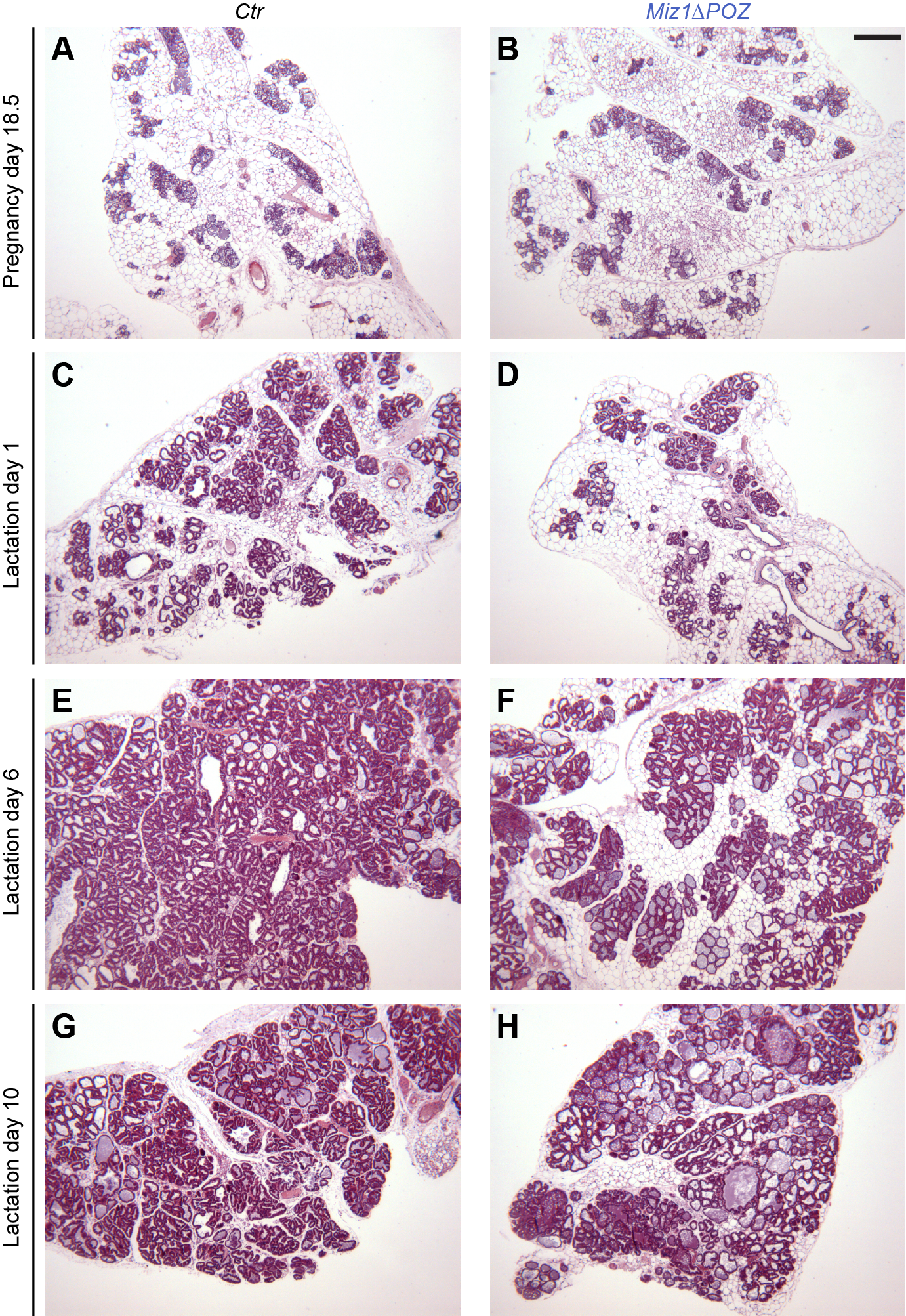

Supplement: Figure S3 — Representative H&E stainings of Ctr and Miz1ΔPOZ animals. In accordance with Miz1 expression levels during mammary development (Fig. 1), first pregnancy day 18.5 samples show similar alveolar density in Ctr and MizΔPOZ animals (A, B; n = 3 per genotype). The reduced alveologenesis phenotype only becomes apparent at lactation day 1 (C, D; at least n = 3 per genotype), obvious at lactation day 6 (E, F and Fig. 2; n = 8 per genotype) and partially rescued by lactation day 10 (G, H; n = 4 per genotype). Scale bar: 300 µm. (TIF) [file pone.0089187.s003.tif]

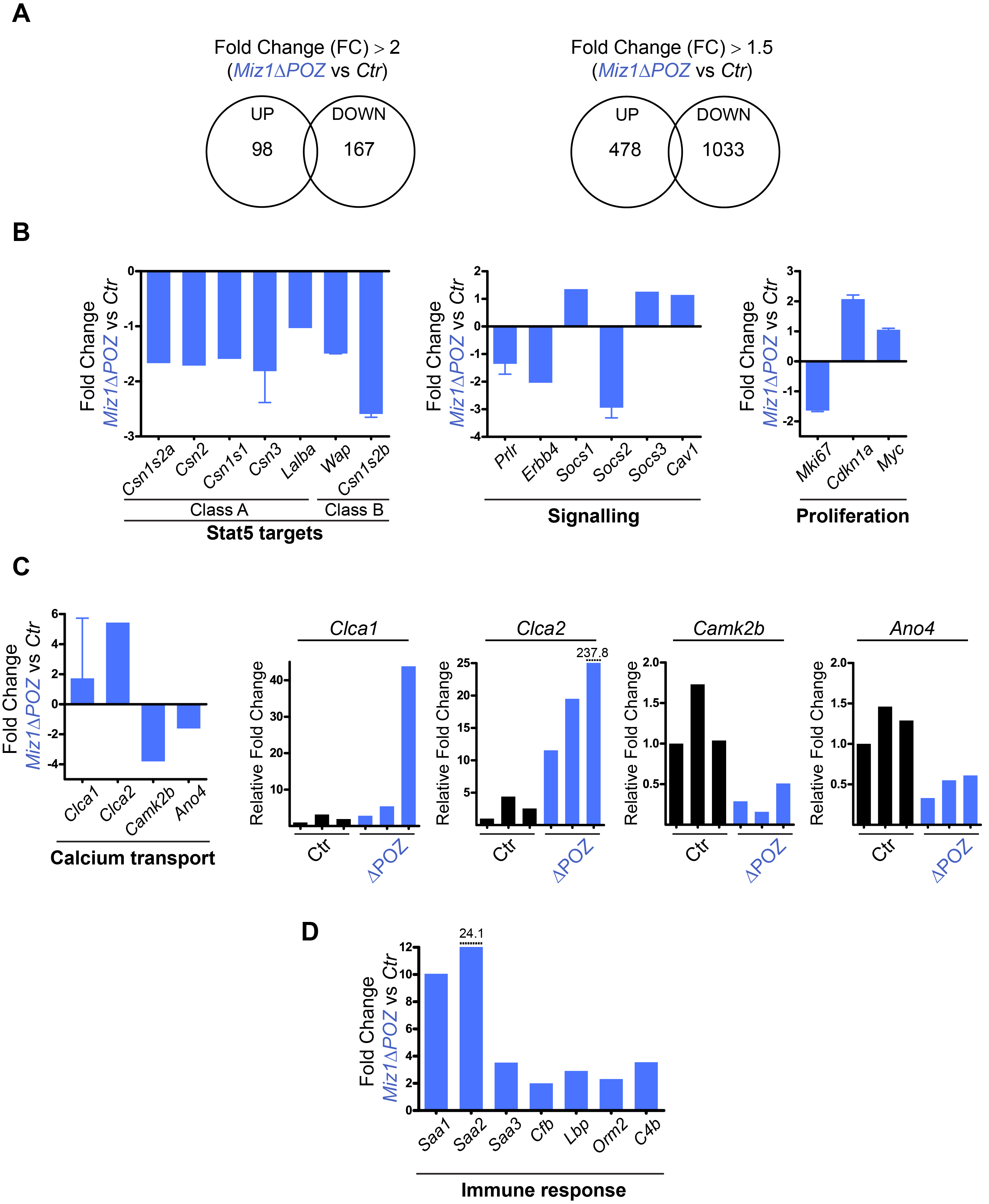

Supplement: Figure S4 — Microarray analysis of lactation day 6 mammary glands. (A) Number of regulated genes in Miz1ΔPOZ mammary glands using different Fold Change (FC) thresholds. Note the increased number of genes down-regulated in Miz1ΔPOZ animals. (B to D) Summary of microarray data, after normalization and filtering, showing the relative expression of different gene sets in Miz1ΔPOZ animals concerning (B) Stat5 target genes, Stat5 signalling and mammary epithelial cell proliferation, (C) calcium transport and (D) immune response. Fold changes were averaged when different values for the same gene were available. See Materials and Methods for experimental details. (TIF) [file pone.0089187.s004.tif]
