# Supplementary material for: Miz1 Deficiency in the Mammary Gland Causes a Lactation Defect by Attenuated Stat5 Expression and Phosphorylation
Source: PLoS One. 2014 Feb 19;9(2):e89187. doi: 10.1371/journal.pone.0089187 (PMC3929623; doi:10.1371/journal.pone.0089187)
Supplement: Table S1 — Summary of all oligonucleotides used for PCR approaches. Primers are provided in 5′→3′ direction. Quantitative PCR primers were designed using the Universal Probe Library Assay Design Center on-line tool (Roche Diagnostics, Mannheim, Germany). (DOC) [file pone.0089187.s005.doc]

**Supplementary Table 1**

| **QUANTITATIVE PCR PRIMERS** | |
| --- | --- |
| ***Ambra1*** | **fw:** GAGCACCCAATTTACCCAGA  **rv:** GATCATCCTCTGGGCGTAGTA |
| ***Ano4*** | **fw:** CGCTTATGACTGGGATCTGATT  **rv:** GCTTCAAACTGGGGTCGTAT |
| ***Camk2b*** | **fw:** AATGCAAGGAGGAAGCTCAA  **rv:** TCCATCTGCTTTCTTGTTGAGTAA |
| ***α casein (s1)*** | **fw:** GACATCTCTCAGGAACTCCACA  **rv:** TCCATAGAATGAATAGAGAGACATGAG |
| ***β casein*** | **fw:** GGTGAATCTCATGGGACAGC  **rv:** TGACTGGATGCTGGAGTGAA |
| ***Cav1*** | **fw:** CCAGGGAAACCTCCTCAGA  **rv:** CCGGATGGGAACAGTGTAGA |
| ***Cdkn1a*** | **fw:** TCCACAGCGATATCCAGACA  **rv:** GGCACACTTTGCTCCTGTG |
| ***Clca1*** | **fw:** CATCTACAAGTGGCAGCGTCT  **rv:** TGCCCCTGCTCTGACATC |
| ***Clca2*** | **fw:** CCAACAGGCTACTGGTGGA  **rv:** TGCTCTGGTCACTGGAGGTA |
| ***Erbb4*** | **fw:** AATGCTGATGGTGGCAAGA  **rv:** CATCACTTTGATGTGTGAATTTCC |
| ***Exoc2*** | **fw:** GGGAGAACCTGGGTACTGGT  **rv:** CCGTGAGGAGGCAATTATGT |
| ***Lrp12*** | **fw:** CAGGCTGGCGTATTTTTCA  **rv:** ATTTCCACAGCGGAACTGAT |
| ***Mki67*** | **fw:** GCTGTCCTCAAGACAATCATCA  **rv:** GGCGTTATCCCAGGAGACT |
| ***Myc*** | **fw:** CCTAGTGCTGCATGAGGAGA  **rv:** TCCACAGACACCACATCAATTT |
| ***Pikfyve*** | **fw:**  GGCCGACTGATCTGGATTC  **rv:**  CCAGCAAATGACCATCAAATAC |
| ***Prlr*** | **fw:** GCAGTGGCTTTGAAGGGTTA  **rv:** CAGACTTGCCCTTCTCTAGCA |
| ***Socs1*** | **fw:** GTGGTTGTGGAGGGTGAGAT  **rv:** CCTGAGAGGTGGGATGAGG |
| ***Socs2*** | **fw:** CGCGAGCTCAGTCAAACAG  **rv:** AGTTCCTTCTGGAGCCTCTTTT |
| ***Socs3*** | **fw:** ATTTCGCTTCGGGACTAGC  **rv:** AACTTGCTGTGGGTGACCAT |
| ***Spast*** | **fw:** CAGCCCTGGGTCCTATCC  **rv:** TTCTCATCTCACTGGCAGACAT |
| ***Stat5a*** | **fw:** AAGATCAAGCTGGGGCACTA  **rv:** CATGGGACAGCGGTCATAC |
| ***Stat5b*** | **fw:** CGAGCTGGTCTTTCAAGTCA  **rv:** CTGGCTGCCGTGAACAAT |

| ***Vamp4*** | **fw:** TGCAAGAGAATATTACAAAGGTAATTG  **rv:** GAAAGCGGTGGCATTATCC |
| --- | --- |
| ***Vps13d*** | **fw:** CTGACTAACCTAGAGCACCAGATCTAT  **rv:** TGTGGTTCCGAAGAGCAAA |
| ***Vps28*** | **fw:** AGCTTCTGTCGCCATCTCC  **rv:** CCCAGGCAGCTATACAGCAC |
| ***WAP*** | **fw:** TGACATGTACACCCCCAGTG  **rv:** CTGGTCACTCCCGACAGG |
| **SEMI-QUANTITATIVE PCR PRIMERS** | |
| ***β actin*** | **fw:** CTAAGGCCAACCGTGAAAAG  **rv:** ACCAGAGGCATACAGGGACA |
| ***β casein*** | **fw:** ACTGTATCCTCTGAGACTG  **rv:** TCTAGGTACTGCAGAAGGTC |
| **GENOTYPING PRIMERS** | |
| ***Cre*** | **fw:** GAACGCACTGATTTCGACCA  **rv:** AACCAGCGTTTTCGTTCTGC |
| ***Miz1*** | **Primer 1:** GTATTCTGCTGTGGGGCTATC  **Primer 2:** GGCTGTGCTGGGGGAAATC  **Primer 3:** GGCAGTTACAGGCTCAGGTG |

Primers are provided in 5´→ 3´ direction. Quantitative PCR primers were designed using the Universal ProbeLibrary Assay Design Center on-line tool (Roche Diagnostics, Mannheim, Germany).
